# A Novel Approach for Visual Speech Recognition Using the Partition-Time Masking and Swin Transformer 3D Convolutional Model

**DOI:** 10.3390/s25082366

**Published:** 2025-04-08

**Authors:** Xiangliang Zhang, Yu Hu, Xiangzhi Liu, Yu Gu, Tong Li, Jibin Yin, Tao Liu

**Affiliations:** 1 The State Key Laboratory of Fluid Power and Mechatronic Systems, School of Mechanical Engineering, Zhejiang University, Hangzhou 310027, China; xlzh@zju.edu.cn (X.Z.); liuxiangzhi@zju.edu.cn (X.L.); yu.gu@zju.edu.cn (Y.G.); 2 Faculty of Information Engineering and Automation, Kunming University of Science and Technology, Kunming 650500, China; hy1786702137@163.com; 3 The Department of Sports Science, College of Education, Zhejiang University, Hangzhou 310027, China; tong.li@zju.edu.cn

**Keywords:** human–computer interaction, lip reading, data augmentation, recognition algorithms, deep learning

## Abstract

Visual speech recognition is a technology that relies on visual information, offering unique advantages in noisy environments or when communicating with individuals with speech impairments. However, this technology still faces challenges, such as limited generalization ability due to different speech habits, high recognition error rates caused by confusable phonemes, and difficulties adapting to complex lighting conditions and facial occlusions. This paper proposes a lip reading data augmentation method—Partition-Time Masking (PTM)—to address these challenges and improve lip reading models’ performance and generalization ability. Applying nonlinear transformations to the training data enhances the model’s generalization ability when handling diverse speakers and environmental conditions. A lip-reading recognition model architecture, Swin Transformer and 3D Convolution (ST3D), was designed to overcome the limitations of traditional lip-reading models that use ResNet-based front-end feature extraction networks. By adopting a strategy that combines Swin Transformer and 3D convolution, the proposed model enhances performance. To validate the effectiveness of the Partition-Time Masking data augmentation method, experiments were conducted on the LRW video dataset using the DC-TCN model, achieving a peak accuracy of 92.15%. The ST3D model was validated on the LRW and LRW1000 video datasets, achieving a maximum accuracy of 56.1% on the LRW1000 dataset and 91.8% on the LRW dataset, outperforming current mainstream lip reading models and demonstrating superior performance on challenging easily confused samples.

## 1. Introduction

Lip reading recognition is a technology that identifies a speaker’s expressed content based on lip movements. This field integrates multiple research areas, including video processing, natural language processing, audio processing, and pattern recognition, demonstrating broad potential across various applications. Visual-assisted speech recognition leverages noise-resistant visual features, such as lip movements, to enhance speech recognition accuracy in noisy environments [1]. In the healthcare domain, this technology aids individuals with speech impairments by facilitating pronunciation training, supporting speech rehabilitation, and providing personalized articulation correction for patients with Parkinson’s disease, thereby improving their speech clarity and communication abilities [2,3,4]. Moreover, lip movement features have been utilized as a novel biometric modality in security applications, such as liveness detection [5].

Despite significant advancements in lip-reading technology in recent years, several technical challenges remain to be addressed. The primary challenge arises from the impact of dynamic variations in ambient lighting conditions, which interfere with the extraction of visual features. Some studies have proposed lip-reading feature extraction methods tailored for varying illumination conditions [6]. Another significant difficulty lies in the visual confusion effect caused by consonants with highly similar lip shapes and tongue positions during pronunciation, such as /m/, /b/, and /p/, as well as their syllabic combinations like pat, mat, and bat [7]. This phenomenon of homophenes significantly increases the complexity of classifier discrimination. Additionally, variations in sequence length further complicate recognition and limit the generalization ability of models, a challenge that has been discussed in prior research [8]. Researchers have proposed various models that demonstrate promising performance in specific aspects to address these issues, but they still exhibit limitations. In the spatial domain, the local receptive field characteristic of CNNs constrains contextual modeling, making it challenging to resolve semantic ambiguities in visually similar phonemes [9]. In the temporal domain, fixed-window 3D CNNs struggle to accommodate the dynamic elasticity of phoneme durations, necessitating the integration of self-attention mechanisms to capture long-range dependencies [10]. Feature space reconstruction methods based on autoencoders, such as multi-band feature fusion strategies [11], can enhance single-frame representations by decoupling deep features. From the feature representation perspective, traditional models’ rigid convolutional kernels are not well suited for capturing the non-rigid nature of lip movements, highlighting the need for deformable convolutions to achieve multi-scale dynamic perception.

To address the aforementioned challenges, this paper proposes a novel lip-reading data augmentation method—Partition-Time Masking (PTM)—to expand and enrich the dataset. A new lip-reading recognition model architecture, Swin Transformer and 3D Convolution (ST3D), was also designed. This model overcomes the limitations of traditional lip-reading recognition models based on ResNet for front-end feature extraction by combining Swin Transformer and 3D convolution strategies. Experiments were conducted on the LAW and LAW1000 datasets to validate the model. Section 2 introduces the proposed data augmentation method (PTM) and lip reading recognition method (ST3D). Section 3 presents the experimental setup and results. Section 4 discusses the performance of the proposed methods. Section 5 concludes this paper.

## 2. Related Works

Researchers have constructed various types of lip reading recognition datasets, providing important support for related studies. Early representative datasets include AVLetters, an English lip-reading dataset recorded by 100 participants, covering the pronunciation of 26 English letters [12]. Subsequently, the AVICAR dataset was proposed, focusing on lip reading recognition in-vehicle environments, containing 10 Arabic numeral samples recorded by 100 speakers in moving vehicles and addressing variations in lighting and background objects [13]. The GRID dataset focuses on phrase-level lip-reading recognition and contains 34,000 short sentences recorded by 34 speakers, each contributing 1000 samples [14]. The OuluVS dataset includes 53 speakers and covers 10 common greeting phrases. Each phrase is repeated 5 times by each speaker, generating a total of 1000 samples, with 5 images from different angles for each sample, thereby increasing the diversity and richness of the samples [15,16]. The LRS2 [17] dataset is a sentence-level audio-free video dataset, and it was compiled by extracting videos from BBC television programs. Similarly, the LRS3 [18] dataset is another sentence-level dataset, created by extracting videos from TEDx talks in a similar manner, with 150,000 sentences sourced from TED programs. The LRW dataset is derived from video clips of over 1000 interviewees in television programs, containing 500 common word categories, each consisting of thousands of samples. The training set of this dataset contains 488,766 samples, the validation set includes 25,000 samples, and the test set consists of 25,000 samples. This dataset is also one of the largest publicly available English isolated word lip-reading datasets to date [19]. The CAS-VAR-W1K (LRW-1000) dataset focuses on word-level recognition in outdoor environments, with 1000 categories and more than 2000 participants, containing 718,018 video samples. The total number of samples exceeds one million Chinese character instances, with each category comprising one or more Chinese characters. This dataset exhibits significant variations in the number of samples per class, video resolution, and lighting conditions, as well as the speaker’s posture, age, gender, and makeup, in order to simulate real-world conditions [20].

Data augmentation methods enhance the performance of network models by increasing the number of samples in the dataset [21]. In lip-reading recognition research, data augmentation methods are divided into two categories: those that do not consider the temporal dimension of the data and those that account for the temporal dimension. Data augmentation methods that do not consider the temporal dimension focus on processing individual frame images of the lip reading data without manipulating the temporal dimension. The study of [22] proposed data augmentation by introducing model prior knowledge, while ref. [23] enhanced the data by randomly cropping portions of the input data. The Mixup method increases the sample size by randomly mixing different training samples [24], and CutMix creates new training samples by overlaying part of one sample onto another [25].

Considering data augmentation methods that consider the temporal dimension, Stafylakis proposed the Word Boundary method, which effectively enhances model performance by incorporating word boundary information [26]. Wang et al. [27], based on SpecAugment [28], introduced the Time Masking data augmentation method, which is widely used in time-series research and is one of the most effective data augmentation techniques to date.

Data augmentation methods that consider the temporal dimension of the data include the Word Boundary method proposed by Stafylakis, which adds indicators containing word boundary information as additional input to the model. These indicators are concatenated with the encoder’s input to form a new input, which is then processed by a temporal model [26]. Wang et al. [27] proposed the Time Masking data augmentation method based on SpecAugment [28], which was initially applied in the field of automatic speech recognition (ASR) and later widely used in time-series research. Time Masking has become one of the most effective data augmentation methods currently available.

Traditional lip-reading recognition models trace back to 1984, when Petajan and his team developed the first lip-reading recognition system, where words were the smallest recognition unit [29]. Subsequently, they introduced the Nostral Tracking method to optimize lip position detection and localization [30]. Researchers such as Goldschen et al. [31] enhanced the spatiotemporal feature extraction of lip movement by employing hidden Markov models (HMMs) [32]. In 2007, Zhao and colleagues introduced a novel spatiotemporal local binary pattern feature extraction method to address the challenges of recognizing isolated English phrases in lip reading [33]. In 2011, Zhao et al. proposed a mathematical mapping approach that projects images from high-dimensional to low-dimensional manifold space, aiming to resolve the issue of lip reading recognition by focusing solely on speech content without considering other speakers [34]. In 2013, Pei et al. proposed a new node-splitting criterion through unsupervised random forest manifold alignment, which effectively captured the motion trajectories of key lip points and demonstrated superior performance across various datasets [35]. In traditional lip-reading recognition research, the active appearance model (AAM) is commonly used. Its core focus is on manually constructing discriminative features to represent lip movements [36].

Deep learning-based lip-reading recognition technology has become mainstream. In 2014, Kuniaki Noda and his team at Waseda University [9] adopted a variant of AlexNet [37], which utilizes deep neural networks to extract feature data from the lips. This study demonstrated the effectiveness of combining deep learning techniques with traditional speech processing methods, providing new perspectives and theoretical support for subsequent lip reading research. In 2017, Assael et al. [38] innovatively introduced connectionist temporal classification (CTC) loss and employed a spatiotemporal convolutional network (TCN) [39] as the front-end feature extraction network, with a bidirectional GRU (Bi-GRU) for temporal classification at the back end. This architecture achieved remarkable results on the GRID dataset, with a recognition accuracy of 95.2%. The success of this model provided important insights for future deep learning-based lip reading research. In the same year, Stafylakis et al. [40] combined TCN [41] with ResNet34 [42] and used Bi-GRU for temporal modeling. In 2020, Petridis et al. [43], inspired by DenseNet [44], proposed a novel network architecture called densely connected temporal convolutional network (DC-TCN). This network effectively utilized the features from shallow networks to address the standard gradient vanishing issue in deep learning training. By combining it with the ResNet18 network for feature extraction, it achieved an accuracy of 88.36% on the LRW dataset and 43.65% on the LRW1000 dataset. In the same year, Ma et al. [41] introduced an innovative network architecture called depthwise separable temporal convolutional network (DS-TCN). This architecture replaced standard convolution with depthwise separable convolution and incorporated ResNet for temporal modeling. On the LRW dataset, DS-TCN achieved a classification accuracy of 46.6%, while, on the LRW1000 dataset, it achieved 88.5% accuracy. In 2022, Petridis and his team proposed a lip-reading recognition model based on DC-TCN and introduced the Time Masking data augmentation method [27]. Their model achieved an astonishing 92.1% classification accuracy on the LRW dataset. That study demonstrated that by refining and optimizing various network components, the performance of lip-reading recognition models could be significantly improved, providing strong guidance and reference for the future development of lip-reading technology.

The innovative applications of Transformer in lip reading have primarily focused on spatiotemporal modeling optimization and multimodal feature fusion. In terms of spatiotemporal modeling, Ma et al. [45] were the first to introduce a hierarchical Transformer into sentence-level Chinese lip-reading tasks. Their approach employed a pinyin-based phoneme separation strategy to better align with the characteristics of Mandarin pronunciation. Wang et al. [46] proposed the 3D convolutional visual transformer (3DCvT), which innovatively integrates dilated convolutional kernels with a bidirectional GRU, enabling joint modeling of short- and long-term motion features. To address the challenge of cross-speaker generalization, Feng et al. [47] designed LipFormer, which incorporates a dual-stream Transformer with cross-attention between visual and landmark features, effectively decoupling lip deformations from facial muscle movements. This method significantly enhances recognition robustness for unseen speakers. Regarding feature optimization, Koumparoulis et al. [48] identified that traditional 3D convolutional models with max-pooling layers tend to suppress the transmission of micro-expression features. To mitigate this issue, their improved EfficientNetV2-Transformer architecture eliminates redundant downsampling operations, preserving fine-grained visual details for enhanced feature extraction.

## 3. Methods and Models

### 3.1. Data Augmentation Methods

Building upon the well-performing data augmentation method, Time Masking [27], in lip reading recognition, we propose a novel data augmentation technique called Partition-Time Masking (PTM). PTM addresses the issue where, after augmenting video clips, only similar portions are retained while other parts are masked. PTM first partitions the input video data into multiple subsequences. Each subsequence is then subjected to the Time Masking operation. To ensure the uniqueness of the mask settings for each subsequence, the starting position and length of the mask for each subsequence are independently and randomly generated. This reduces the similarity between different sequences, enhancing the model’s ability to handle similar samples. Finally, all subsequences are concatenated in sequence to produce the augmented dataset. The specific operation steps of the PTM method are shown in Algorithm 1.
**Algorithm 1** PTM operation steps  1:**Input:**  2:    Input data *X* and divide it into *k* subsequences, each containing $T_{k}$ consecutive frames.  3:    $X_{n}$ represents the *n*-th subsequence.  4:**Step 1: Divide the data into subsequences**  5:    $T_{k}=\left\lfloor \frac{\left|X\right|}{k}\right\rfloor$  6:    $X_{n}\left[0:T_{k}\right]=X\left[\left(n-1\right)T_{k}:nT_{k}\right]$  7:**Step 2: Compute mask length and intermediate variable**  8:    $\tau =\alpha T_{k}$  9:    $\delta ,t_{0}∼U\left(0,\tau \right)$ 10:**Step 3: Calculate mask starting position** 11:    $t_{1}∼U\left(0,T_{k}-\delta \right)$ 12:**Step 4: Apply masking operation** 13:    Apply masking operation to the *k*-th subsequence of *X* starting from $t_{1}$ for $t_{0}$ consecutive frames 14:    $X_{n}\left[t_{1}:t_{1}+t_{0}\right]=X_{n}.mean\left(\right)$ 15:**Step 5: Repeat for all subsequences** 16:    Repeat Steps 2–4 for *k* times. 17:**Step 6: Concatenate subsequences** 18:    Concatenate all subsequences to obtain augmented data *Y* 19:    $Y=vstack(X_{1},\ldots ,X_{k})$ 20:**Output:** 21:    Output the augmented data *Y*

We designed several enhancement strategies, incorporating varying mask values and different numbers of subsequences. The mask values were categorized into the original input average frame (R) and the subsequence average frame (P). The number of subsequences was set to 2, 3, or 5. These combinations resulted in the following strategies: R-MaskDouble (RD), R-MaskThree (RT), R-MaskFive (RF), P-MaskDouble (PD), P-MaskThree (PT), and P-MaskFive (PF).

### 3.2. Lip Reading Recognition Model

We improved the mainstream lip-reading recognition model, which is based on the ResNet18 front-end feature extraction network [10,42], by combining 3D convolution with the Swin Transformer, resulting in a novel lip-reading recognition model (ST3D). This method first utilizes 3D convolution to extract the spatiotemporal features of lip movements, and it then further extracts and integrates these features effectively through the Swin Transformer network. Finally, the extracted feature information is processed by DC-TCN and Softmax, where temporal modeling and classification probabilities are computed before the final output.

**Figure 1:**
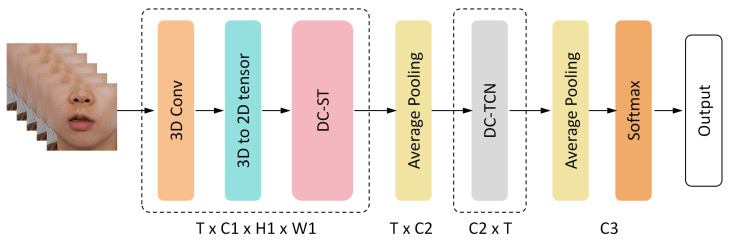
ST3D model architecture.

The ST3D model adopts an end-to-end structure, as shown in Figure 1. The front-end structure utilizes a pyramid-like layout integrating 3D and 2D convolutional layers. The model incorporates a combination of 3D convolution and a Swin Transformer variant module to construct a densely connected Swin Transformer (DC-ST), which more effectively captures the key visual features of lip dynamics. At the model’s back end, a densely connected temporal convolutional network (DC-TCN) is used for temporal modeling.

#### 3.2.1. 3D Convolutional Module

First, a 3D convolutional module is applied to process the input video, extracting the spatiotemporal features in the video data. This effectively captures the dynamic movements and subtle changes in the lips. After passing through a 3D-to-2D conversion layer, the three-dimensional video data are transformed into two-dimensional image data using a dimensional transposition operation. This process preserves the crucial temporal information in the video while generating two-dimensional image data with dimensions of $H_{1}\times W_{1}$. The specific operation steps are shown in Algorithm 2.
**Algorithm 2** 3D convolution operation steps  1:**Input:**  2:    Input video data $X\in R^{BZ\times C\times L\times H\times W}$  3:**Output:**  4:    Transformed data $X^{\prime }$  5:**Procedure TRANSPOSE_AND_RESHAPE**()  6:    Extract the shape of *X*: BZ,C,L,H,W=X.shape  7:    **Step 1: Transpose Operation**  8:    Swap the first and second dimensions of *X*  9:    X=X.transpose(1,2) 10:    **Step 2: Reshape Operation** 11:    Reshape *X* into the shape (BZ×L,C,H,W) 12:    $X^{\prime }=X.reshape\left(BZ\times L,C,H,W\right)$ 13:**End Procedure** 14:Return the transformed data $X^{\prime }$

Let *X* represent the input video data, where BZ, *C*, *L*, *H*, and *W* denote the batch size, the number of channels, the sequence length, the image height, and the image width, respectively. The transpose operation refers to swapping the first and second dimensions of the data, while the reshape operation is used to transform the data into the specified shape.

The 3D convolution module consists of a 3D convolution layer, a batch normalization (BN) layer, and a ReLU activation function. The input data are passed through the 3D convolution layer, which learns the spatial–temporal features of the data. Simultaneously, the size of each image in the sequence is reduced by half, improving computational efficiency. The kernel size of the convolution layer (in terms of time/width/height) is 3×5×5, with a stride of 1×2×2. The data are then normalized through the BN layer, followed by applying the ReLU, and then the result. No compression of the time dimension is applied in the 3D convolution module as the goal is to capture subtle temporal variations, better understand the contextual relationships in time, and avoid losing information too early.

#### 3.2.2. DC-ST Module

Based on the data processed by the 3D convolution module, the DC-ST module is used to extract image features further, gradually reducing the spatial resolution of the images while increasing the number of channels. This process enables the network to capture more profound and more subtle visual information, allowing it to learn more complex and richer image features. Subsequently, the features are further processed through an average pooling layer, reducing the height and width of each feature map to 1, thus achieving dimensional fusion and simplification. The final output is a one-dimensional temporal sequence data with shape $\left(C_{2}\right)$.

**Figure 2:**
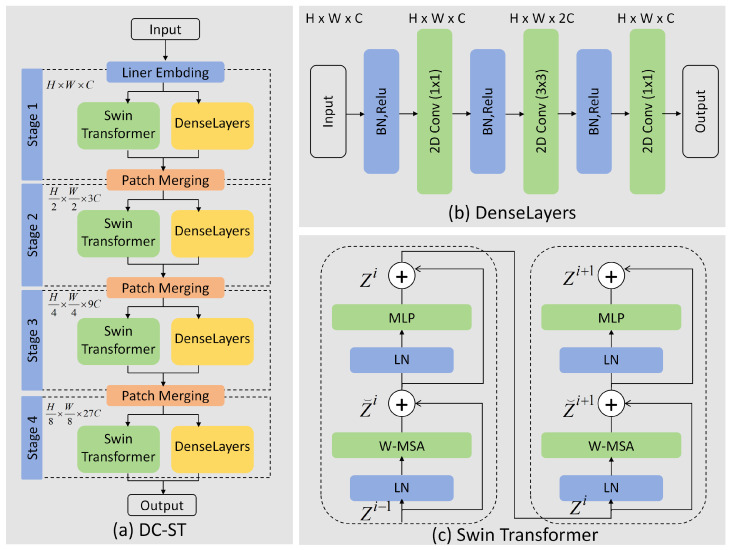
Framework of each module. (**a**) Diagram of the DC-ST module structure; (**b**) diagram of the DenseLayers module structure; and (**c**) diagram of the Swin Transformer module structure.

The structure of the DC-ST module is shown in Figure 2a. This module consists primarily of three components: Swin Transformer, Denselayers, and Patch Merging. The architecture of DenseNet inspires the core of the DC-ST module. Each DenseBlock is composed of several Denselayers, and, within the module, Denselayers are connected with Swin Transformer via residual connections to increase feature richness. The Patch Merging layer functions similarly to a pooling layer, decreasing the spatial resolution of feature maps while tripling the number of channels. This design enables feature reuse and expansion of the channel dimension. Bypassing the input from the previous layer to the next layer and concatenating the data increases the channel number, thus enriching the feature representation and expanding the network’s capacity to process information. This addresses the potential issue of insufficient channel numbers in the Swin Transformer when processing lip reading data. Additionally, this design optimizes information and gradient flow as the closely connected layers improve information propagation and gradient flow between the network layers.

Denselayers and Swin Transformer are two key components of the DC-ST module, each with a unique internal structure that optimizes the performance of the lip-reading recognition model. Each Denselayer consists of three 2D convolutional layers. The first convolutional layer employs a kernel size of 1×1, primarily to fuse features from the previous layer while doubling the number of output channels to extract more complex and rich feature information. The second convolutional layer uses a kernel size of 3×3 and primarily focuses on learning the spatial features within the image, maintaining the same number of channels as the previous layer. The third convolutional layer, with a kernel size of 1×1, aims to reduce the number of channels in the feature map, optimizing computational efficiency while preserving key feature information, as illustrated in Figure 2b.

The Swin Transformer module effectively extracts and processes input features by combining LN normalization layers, self-attention mechanisms, and MLPs, achieving the fusion of local and global information. In the first stage, the input is processed through an LN normalization layer, enhancing the training stability and increasing the inputs’ robustness. Then, a window-based self-attention calculation (W-MSA) is performed within fixed-sized windows to capture local features. The output is further processed by an LN layer and a multi-layer perceptron (MLP) to introduce non-linearity and to refine the features. In the next stage, a sliding window mechanism is used for self-attention calculation (SW-MSA), where the spatial shift of the window helps capture broader contextual information. The output is then passed through another LN normalization layer and MLP, further learning the feature information. Finally, the output of the MLP is processed through the last LN normalization layer, completing the computation flow. The structure is illustrated in Figure 2c. In each stage, the data dimensions remain unchanged, ensuring the effective extraction and processing of features while maintaining numerical stability through layer normalization. The output of each stage can be used for the subsequent stage or, in the final stage, for specific visual tasks. The Swin Transformer can efficiently handle image data through this hierarchical and window-based approach while maintaining computational efficiency.

The outputs of the Swin Transformer and Denselayers are concatenated along the channel dimension before being fed into the Patch Merging layer, which halves the spatial size while tripling the number of channels. The process then moves to the next stage. In DC-ST, there are four stages, each of which are formed by stacking the aforementioned modules multiple times, followed by an average pooling layer for the final output.

#### 3.2.3. Back-End Module

The back-end temporal modeling of ST3D primarily utilizes the DC-TCN network. This network selectively retains and forgets feature sequence information through multi-layer, dilated, and causal convolutions. The DC-TCN network first receives the feature output from the ST3D module and then performs temporal modeling. In this step, the time and channel dimensions of the input are first transposed, with the time dimension placed last to enable the network to capture dynamic changes along the time axis. Finally, a second average pooling layer performs temporal averaging on the one-dimensional time series data, resulting in an output vector with dimensions of $\left(C_{3}\right)$. The output is then fed into the SoftMax layer for classification, and the final classification result is calculated as follows, where *k* denotes the number of classes and $X_{i}$ represents the output probability for the *i*-th sample’s classification:

(1) $$P_{i}\left(X\right)=\frac{e^{x_{i}}}{\sum_{k}e^{x_{i}}}.$$

## 4. Experiments and Results

### 4.1. Data Preprocessing

This study conducted experiments on the LRW and LRW1000 datasets. Prior to experimentation, preprocessing steps were applied to the datasets as follows.

LRW Dataset: First, the RetinaFace tool [24] is used to detect faces in the videos. Then, 25 consecutive frames containing the speaking portion (approximately 1 s) are extracted from each video, and the remaining frames are discarded. If a sample contains fewer than 25 valid frames, padding frames with a value of 0 are added to ensure consistency in the number of input frames [25]. Next, each frame is cropped from the region $\left(x_{1},y_{1},x_{2},y_{2}\right)=\left(80,116,175,211\right)$ to obtain lip images of size 96 × 96 [22]. These images are further randomly cropped to 88 × 88 and subjected to random horizontal flipping. Finally, the RGB images are converted to grayscale before being fed into the model.

LRW1000 Dataset: The faces in the videos are detected, and all of the frames containing the speaker’s lip movements are extracted (the number of frames is variable). These frames are cropped to 128 × 128 pixel lip images, which are then resized to 88 × 88. The images are randomly horizontally flipped, and the RGB data are then converted to grayscale for model input.

Dataset Splitting: The LRW dataset consists of 488,766 training samples, with 25,000 samples for both the validation and test sets. The LRW1000 dataset contains 629,366 training samples, 63,381 validation samples, and 52,441 test samples.

### 4.2. Experimental Results of Data Augmentation Methods

To validate the performance enhancement of the Partition-Time Masking (PTM) method on lip reading recognition models, this study selected three commonly used models in lip reading research: DC-TCN, MS-TCN, and Bi-GRU. All experiments were conducted under the same software and hardware environment. The specific experimental configuration was as follows: RTX 3090 GPU, Windows 10 operating system, CUDA version 11.1, TorchVision version 0.9.0, and PyTorch version 1.8.0. The initial learning rate was 0.0003, and the number of training epochs was 80. The DC-TCN and MS-TCN networks were trained on the LRW dataset, while the Bi-GRU network was trained on the LRW1000 dataset. Additionally, a control group using the Word Boundary method was included. The experimental results with different data augmentation methods are shown in Table 1.

An analysis of the experimental results, which is presented in Table 1, revealed that, when the data are divided into two sub-sequences, the performance of the three network models—DC-TCN, MS-TCN, and Bi-GRU—improved in all experimental groups except for the DC-TCN with the word boundary group. The performance improvement in these groups surpassed that of the baseline group with Time Masking augmentation.

We analyzed the accuracy decline observed in the DC-TCN with the Word Boundary experiment. It was found that, during the early stages of training, the accuracy and loss of this group fluctuated, whereas other models stabilized in the last few rounds of training. This suggests that the model might not have been fully trained due to insufficient training epochs. To verify this hypothesis, we retrained, using different numbers of training epochs, the DC-TCN model with the Word Boundary and RD strategies and the DC-TCN model with the Word Boundary method and Time Masking. The experimental results are shown in Table 2, where it is evident that the optimal number of training epochs was 90.

Further experiments were conducted to determine the impact of the number of subsequences and mask selection in the Partition-Time Masking method on the final network performance and to identify the optimal strategy combination. The DC-TCN network was trained on different enhancement strategies for the LRW dataset. The experimental accuracy for RD was 90.03%, RT 89.82%, RF 89.61%, PD 90.08%, and PT was 89.94%. The results indicate that network performance improves progressively as the number of subsequences increases from 2 to 3; however, when the number of subsequences is increased to 5, overfitting may occur, leading to a reduction in network performance. Compared to using the average frame of the original input as the mask value, selecting the average frame of each subsequence as the mask value better enhances the model’s performance. The optimal strategy currently is RD, where the number of subsequences is three, and the mask value for each subsequence is the average frame of that subsequence. In summary, the Partition-Time Masking method significantly improves network performance more than the Time Masking method.

### 4.3. Experimental Results of the Lip Reading Recognition Models

The experiment was conducted using the LRW and LRW1000 datasets. The primary goal of this experiment was to evaluate the accuracy of the models in the lip reading recognition task. Thus, the highest output accuracy of each model was selected as the key performance metric. The accuracy was calculated as the ratio of correctly predicted samples to the total number of samples, providing a direct quantitative indicator of model performance. Experimental configuration: the CPU used was Intel(R) Core(TM) i9-10900k; and the GPU was NVIDIA GeForce RTX3090, which had 32 GB of RAM. The software environment included Python 3.8 and PyTorch 1.10.1. The parameter settings during the model training process are as follows: learning rate was 0.0004, dropout rate was 0.3, training epochs was 100, optimizer was adamW, and the batch size was 32.

To verify the generalization ability and scalability of the lip reading model ST3D, this study designed three variants of ST3D with different parameter configurations: ST3D-I, ST3D-II, and ST3D-III. The main differences between these variants lie in the number (N) of Swin Transformer blocks and the number (H) of attention heads in each stage. In ST3D-I, N is set to 1, 1, 3, 1 and H is set to 1, 3, 6, 9. In ST3D-II, N is set to 2, 2, 6, 2 and H is set to 3, 3, 6, 12. In ST3D-III, N is set to 2, 2, 9, 2 and H is set to 3, 9, 12, 18. These configurations were used to explore the scalability of the ST3D architecture. These models were experimentally tested on the LRW and LRW-1000 lip reading datasets with the other parameters held constant. On the LRW dataset, the accuracy of ST3D-I was 90.5%, ST3D-II was 91%, and ST3D-III was 91.8%. On the LRW-1000 dataset, ST3D-I achieved an accuracy of 55.2%, ST3D-II 56.1%, and ST3D-III 55.7%. The experimental results show that ST3D-III achieved the best recognition accuracy on the LRW dataset, while ST3D-II performed best on the LRW-1000 dataset. Therefore, different parameter configurations can be selected depending on the specific task requirements.

This study also explored the model’s optimization strategies and validated their impact on lip reading task performance through ablation experiments. The optimization strategies include the use of word boundary methods to add additional input dimensions during the construction of the temporal model, applying Mixup, Cutmix, Time Masking, and Partition-Time Masking methods during the data preprocessing phase, as well as label smoothing techniques. The experimental results are shown in Table 3. With the cumulative application of these strategies, the model’s accuracy gradually improved on both the LRW and LRW1000 datasets. Among these, the inclusion of Mixup and Cutmix methods led to a significant accuracy boost, with improvements of 0.7% and 0.3% on LRW, respectively. Subsequently, the label smoothing mechanism further optimized the model’s performance. The introduction of word boundary information significantly improved recognition accuracy. Finally, at the last stage, the Partition-Time Masking data augmentation strategy showed more pronounced performance improvements compared to the Time Masking method, achieving up to a 0.4% increase.

## 5. Discussion

This paper first proposes a data augmentation method, PTM. It uses three models—DC-TCN, MS-TCN, and Bi-GRU—as experimental subjects to validate the data augmentation performance using the LAW and LAW1000 datasets. During the experiments, we added Word Boundary and Time Masking as control groups. Based on the experimental results, we found that the PTM model performed better in most experimental groups and outperformed the Time Masking model. The highest accuracy achieved on the LAW dataset was 91.84%, while, on the LAW1000 dataset, the highest accuracy was 55.7%.

However, in one experimental group of the DC-TCN model, the accuracy using the proposed PTM model was significantly lower than that of the Time Masking model. We conducted an in-depth analysis of the training process of the DC-TCN network, observing the changes in loss and accuracy during each training round. This may be due to an insufficient number of training epochs. We decided to increase the number of training epochs to verify this hypothesis to improve the model performance. In subsequent experiments, we set the training epochs to 90 and 100 as control groups, which are commonly used in lip reading research. We retrained the model using the DC-TCN network with the Word Boundary and RD strategies. The experimental results indicated that the highest accuracy of our proposed data augmentation method was achieved at 90 epochs, reaching 92.15%. We also explored the impact of different strategies on the performance of the data augmentation method. The experimental results showed that the best strategy was PD, where the number of subsequences is 3, and the mask value for each subsequence is the average frame of the respective subsequence.

This paper proposes a new lip-reading recognition model, ST3D. We compared the ST3D model with several commonly used lip-reading recognition models in the literature, and the experimental results are shown in Table 4. As seen from the table, compared to the current lip-reading recognition networks that use ResNet18 as the front-end feature extraction network, the application of the Swin Transformer structure in this study yielded better results. Integrating the Swin Transformer architecture into lip-reading models enhances their ability to process information, particularly by addressing the limitation of traditional CNNs, which primarily focus on local features. The Swin Transformer’s flexibility allows it to adaptively adjust the receptive field size, effectively capturing rich information ranging from fine-grained details to global contexts. This enables the model to recognize subtle lip movements and understand their significance within facial expressions and contextual information. Moreover, the self-attention mechanism embedded in the Swin Transformer is particularly advantageous for lip reading. It prioritizes key regions crucial for recognition, thereby improving the extraction and emphasis of important features. This is especially critical for handling the non-rigid nature of lip movements, which requires the model to detect fine details and comprehend and interpret dynamic changes in motion. The model achieved a recognition accuracy of 91.8% on the LRW dataset, which is comparable to the best current recognition results, and a recognition accuracy of 56.1% on the LRW-1000 dataset, surpassing the best existing recognition results. The experimental results validate the effectiveness of ST3D, demonstrating that the model achieved good results on both significant datasets and showed certain advantages in feature extraction.

To investigate the advantages of the ST3D model in addressing visual ambiguity and the correlation between the lips and other facial features, we selected the most easily confused samples (with low recognition accuracy) from both the LRW and LRW1000 datasets. These samples were chosen based on a comprehensive analysis of the results from various models. The changes in the confusion rates of the 10 most easily confused samples from the LRW and LRW1000 datasets are listed in Table 5 and Table 6, respectively. As shown, these 10 easily confused samples are very similar in both spelling and pronunciation. The confusion rate is the proportion of samples in a category incorrectly identified as other categories out of the total number of samples in that category. From Table 5, we can see that, in the LRW dataset, only 2 out of the 10 easily confused samples of the ST3D model had higher confusion rates than the DC-TCN model, one sample had the same confusion rate as DC-TCN, and the remaining seven samples had lower confusion rates than DC-TCN. Table 6 shows that, in the LRW1000 dataset, 5 of the 10 easily confused samples of the ST3D model had higher confusion rates than DC-TCN, while the remaining 5 samples had lower confusion rates than DC-TCN. Overall, ST3D was found to be more effective in handling visual ambiguities on the LRW dataset; however, its performance in addressing visual ambiguities did not improve on the LRW1000 dataset.

**Figure 3:**
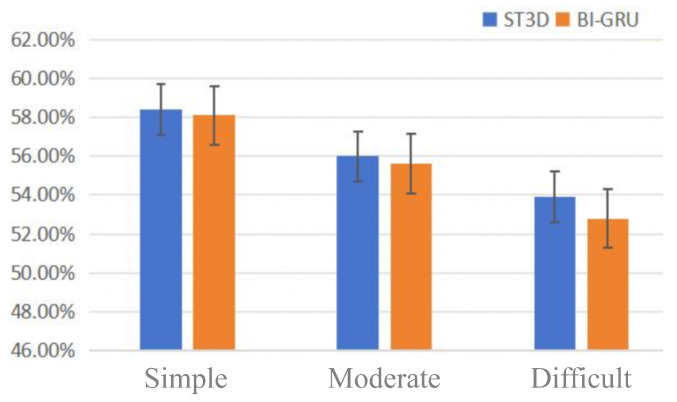
The model performance on different sequence lengths.

We further categorized the LRW1000 dataset into three difficulty levels based on the length of input sequences (i.e., the number of video frames). Specifically, sequences with lengths ranging from 15 to 30 frames were classified as simple, those between 5 and 15 as moderate, and those with fewer than 5 frames as difficult. We compared the performance of the ST3D model and Bi-GRU models’ performance across samples of different difficulty levels with the results presented in Figure 3. The experimental results indicate that the ST3D model consistently outperformed the MS-TCN model across all difficulty levels, and both models exhibited performance improvements as sequence length increases. Further analysis revealed a positive correlation between the recognition accuracy and the amount of information contained within a sequence. Shorter sequences may lack sufficient information to support accurate recognition and are more susceptible to external noise. Moreover, the training dataset showed an imbalanced distribution of sequence lengths, with significantly more long-sequence samples than short-sequence ones. This imbalance leads models to focus more on long-sequence samples during training. Additionally, compared to the MS-TCN model, the ST3D model demonstrated superior temporal modeling and feature extraction capabilities, enabling it to perform better when processing samples of the same difficulty level.

While our approach significantly improves visual speech recognition, some limitations remain. Dataset Scope: This study focused on word-level recognition using LRW and LRW1000, which serve as challenging benchmarks. However, they do not encompass sentence-level lip-reading, which is crucial for real-world applications. Future work should extend our method to datasets like LRS2 and LRS3 to evaluate its generalizability in sentence-level tasks. Input Scale Analysis: We analyzed the impact of sequence length on recognition accuracy but did not explicitly investigate the variations in input scales (e.g., different spatial resolutions of lip regions). Examining their influence on feature extraction and model performance could provide valuable insights for optimization. Computational Efficiency: The integration of Swin Transformer and 3D convolution strengthens spatial–temporal feature learning but increases computational cost. Future research should explore lightweight architectures or knowledge distillation to improve efficiency without compromising accuracy. Addressing these limitations will further enhance the robustness and applicability of our approach.

## 6. Conclusions

This paper proposes a data augmentation method, PTM, which first partitions the input video data into multiple subsequences and then applies Time Masking to each subsequence individually. Using the DC-TCN model for training and validation on the LAW dataset, the highest accuracy can reach 92.15%. In addition, we propose a lip-reading model, ST3D, which introduces the Swin Transformer structure into the lip-reading model, enhancing the model’s information processing capability. This addresses the limitation of traditional CNNs, which focus on local information and help capture and analyze the complex dynamic features in lip-reading recognition, thereby improving overall recognition efficiency and accuracy. On the LAW1000 dataset, the highest accuracy can reach 56.1%, and, on the LAW dataset, the highest accuracy can reach 91.8%. However, there are still areas for improvement, and the recognition accuracy can be further enhanced. In the next step, we will improve our lip-reading recognition algorithm to boost the recognition rate.

The data augmentation method and lip-reading model we propose has broad application potential. We can enhance the robustness of lip-reading recognition in complex scenarios, such as assisting individuals with hearing impairments in noisy environments or multi-party conversations or in applying it to silent command recognition in public security fields. These advancements can extend the boundaries of human–computer interaction applications. However, the research still has limitations. For instance, the model relies on detecting high-quality lip regions, and if there are occlusions or extreme lighting changes in the video, the recognition performance may degrade significantly. Although the hybrid architecture of the Swin Transformer and 3D convolution enhances spatiotemporal feature fusion, its large number of parameters makes it unsuitable for real-time applications. Future research will focus on model lightweight and real-time performance improvement, such as adopting parameter compression techniques from compact 3D convolutions to designing more efficient hybrid architectures and exploring multimodal deep fusion—such as combining audio residual signals or facial expression features—to improve recognition accuracy. Additionally, efforts will be made to enhance the model’s robustness and generalization ability, such as, for example, by developing adversarial training strategies to address the interference from lighting and occlusion. Finally, practical application scenarios will be explored to validate the model’s usability in medical assistance and security monitoring while optimizing real-time interaction experiences.

## Figures and Tables

**Table 1:** Performance of the models with different augmentation methods.

Model	Dataset	Augmentation Method			Accuracy
		Word Boundary	Time Masking	RD	
DC-TCN	LRW	✔			92.11%
		✔		✔	91.84%
			✔		89.61%
				✔	90.03%
MS-TCN	LRW	✔	✔		88.88%
		✔		✔	88.91%
Bi-GRU	LRW1000	✔	✔		55.5%
		✔		✔	55.7%

**Table 2:** The impact of the model enhancement strategies and training epochs on accuracy.

Model	Enhancement Strategy	Training Epochs	Accuracy
DC-TCN	Word Boundary + Time Masking	80	92.11%
		90	92.08%
		100	91.91%
DC-TCN	Word Boundary + RD	80	91.84%
		90	92.15%
		100	91.96%

**Table 3:** Performance of the ST3D model after adding data augmentation methods.

Adjust	LRW	LRW1000
Baseline Model	86.4%	53.6%
+Mixup	87.1%	54.5%
+Cutmix	87.4%	54.8%
+Label Smoothing	88.6%	55.1%
+Word Boundary	89.7%	55.5%
+Time Masking	91.5%	55.4%
Partition-Time Masking	91.9%	55.7%

**Table 4:** Comparison with existing model performance.

Work	Model		Dataset	
	Front-End	Back-End	LRW	LRW1000
Weng [10]	Tow-Stream	Bi-LSTM	84.1%	-
Ma [49]	3D + ResNet-18	MS-TCN	87.7%	43.2%
Feng [22]	3D + SE + ResNet-18	Bi-GRU	88.8%	55.7%
Kim [50]	3D Conv + ResNet-18	Bi-GRU	85.4%	50.8%
Ma [27]	3D Conv + ResNet-18	DC-TCN	92.0%	-
Our work	ST3D	DC-TCN	91.8%	56.1%

**Table 5:** The confusing 10 samples in the LRW dataset.

Label	Predicted Result	DC-TCN Confusion Rate	ST3D Confusion Rate
GIVING	LIVING	20%	18%
SERIOUS	SERIES	20%	17%
SPEND	SPENT	20%	19%
BENEFITS	BENEFIT	18%	19%
WANTS	WANTED	18%	16%
WANTED	WANTS	16%	13%
PERSONAL	PERSON	16%	16%
DIFFERENCE	DIFFERENT	16%	17%
COUNTRY	COUNTRIES	16%	14%
BENEFIT	BENEFITS	16%	15%

**Table 6:** The confusing 10 samples in the LRW1000 dataset.

Label	Predicted Result	DC-TCN Confusion Rate	ST3D Confusion Rate
Zhang	Zhan	30%	28%
Zheng Chang	Zeng Chan	20%	17%
Zheng	Zhen	20%	29%
Bang	Ban	18%	19%
Zhi	Zi	18%	16%
Zhuan	Zhuang	56%	33%
Zhong He	Zhong He	13%	16%
Zhu He	Zu He	12%	17%
Zou	Zhou	16%	24%
Tong Bao	Tao Bao	36%	15%

## Data Availability

The data used in this article come from two sources: the LRW dataset provided by BBC Research & Development [available from https://www.robots.ox.ac.uk/~vgg/data/lip_reading/lrw1.html], and the LRW-1000 dataset provided by the VIPL group at the Institute of Computing Technology, Chinese Academy of Sciences [available from https://vipl.ict.ac.cn/resources/databases/].
